# Metabolomic profiling and biomarker identification for early detection and therapeutic targeting of doxorubicin-induced cardiotoxicity

**DOI:** 10.3389/fcell.2025.1543636

**Published:** 2025-04-10

**Authors:** Jingjing Ding, Xianzhen Feng, Zhongqing Xu, Hong Xu

**Affiliations:** ^1^ Department of Oncology, The First Affiliated Hospital of Soochow University, Suzhou, China; ^2^ Department of General Practice, Tongren Hospital, Shanghai Jiao Tong University School of Medicine, Shanghai, China

**Keywords:** cardiotoxicity, anticancer, metabolomics, biomarker, doxorubicin

## Abstract

**Background:**

Doxorubicin (DOX) is a widely used chemotherapeutic agent known for its efficacy against various cancers, but its clinical application is often limited by its cardiotoxic effects. The exact mechanisms of DOX-induced cardiotoxicity remain unclear, requiring further investigation. Early diagnosis is essential to enhance the quality of life and prognosis for patients with malignancies. This study aims to identify biomarkers and therapeutic targets for DOX cardiotoxicity.

**Methods:**

Heart tissue samples from 20 DOX-treated cardiotoxic mice and 19 normal controls were analyzed using liquid chromatography-mass spectrometry (LC-MS). Multivariate statistical analysis identified differential metabolites. Key metabolites were assessed using a random forest algorithm, and ROC curves evaluated diagnostic value. H9C2 rat cardiomyoblast cells were cultured to investigate the protective effects of these metabolites.

**Results:**

Among 291 metabolites, significant differences emerged between cardiotoxic and normal mice. Five metabolites-4-hydroxy-valeric acid, 2-methylbutanoic acid, traumatic acid, PI (18:2 (9Z, 12Z)/0:0), and MIPC (t18:0/24:0 (2OH))-showed diagnostic potential. ROC analysis indicated excellent value for 4-hydroxy-valeric acid and PI (18:2 (9Z, 12Z)/0:0) and high discriminatory power for 2-methylbutanoic acid (AUC = 0. 99). Pathway analysis highlighted glycosylphosphatidylinositol-anchor biosynthesis, unsaturated fatty acids biosynthesis, pantothenate and CoA pathways, among others, associated with DOX-induced cardiotoxicity. In addition, we found that the differential metabolite Cer (d18:0/12:0) can improve DOX-induced myocardial cell damage and inhibit apoptosis-related protein expression at the cellular level.

**Conclusion:**

Heart tissue metabolomics with LC-MS identified critical metabolites and pathways associated with DOX cardiotoxicity, suggesting biomarkers for early diagnosis and potential therapeutic targets to mitigate DOX-related cardiotoxicity and improve clinical outcomes.

## Background

In recent years, as early diagnosis and treatment strategies for cancer continue to advance, the survival period of cancer patients has been extended. However, the incidence and mortality of complications caused by anticancer treatment have also increased annually, with cardiovascular disease being one of the most common adverse reactions triggered by anticancer therapy (Ortega et al., 2021). Consequently, the field of Onco-Cardiology has emerged. Anthracycline chemotherapy drugs are a class of highly effective tumor chemotherapy drugs widely used in the medical treatment of various malignant tumors. However, their dose-dependent cardiotoxicity limits their clinical application (Volkova and Russell, 2011).

DOX is a highly effective anthracycline chemotherapy drug, and studies show that 33% of cancer survivors die from cardiac complications, with 51% having used anthracycline drugs (Henriksen, 2018). Despite their impressive effectiveness against tumors, clinicians need to be cautious about the potential cardiotoxic effects of these drugs. Research has indicated that anthracyclines, especially DOX, can lead to cardiac dysfunction and, in some cases, even irreversible heart failure (Bayles et al., 2023). It is essential to understand the mechanisms behind anthracycline-induced cardiotoxicity and to effectively monitor and manage these side effects to enhance patients’ quality of life (Sun et al., 2023). Although decades of research have led to significant progress in understanding the molecular and pathophysiological mechanisms of cardiotoxicity caused by anticancer drugs, several challenges still persist. These include the absence of sensitive predictive biomarkers, a shortage of effective therapeutic options, and an incomplete understanding of the underlying mechanisms. As a result, there is a pressing need for early diagnostic methods and the discovery of new therapeutic targets to prevent and manage DOX-induced cardiotoxicity (Zhang et al., 2023).

The cardiotoxicity of anthracycline drugs primarily manifests as changes in myocardial cell morphology and heart function, including myocardial fiber loss, cytoplasmic vacuolation of myocardial cells, disappearance of organelles, nuclear degeneration, and abnormal electrocardiograms (such as QT interval prolongation, supraventricular arrhythmias, ventricular arrhythmias), cardiac impairments such as diastolic and systolic dysfunction of the left ventricle, as well as dysfunction of the right ventricle, and severe cases may result in congestive heart failure (Swain et al., 2003; Chatterjee et al., 2010). Studies have shown that anthracycline chemotherapy drugs may cause a marked decrease in left ventricular ejection fraction (LVEF). Among early breast cancer patients over 60 years old receiving anthracycline chemotherapy, up to 33% experienced a LVEF decline of 10% or more (Hamirani et al., 2016). The cardiotoxicity caused by anthracycline chemotherapy drugs often necessitates the premature termination of chemotherapy, increasing the risk of cancer recurrence.

Metabolomics research offers hypotheses on disease mechanisms and drug treatment effects, opening new research areas for better understanding treatment processes in target populations (Puchades-Carrasco and Pineda-Lucena, 2015). Metabolomics has revealed critical insights into the mechanisms underlying DOX-induced cardiotoxicity, identifying disrupted metabolic pathways and potential therapeutic targets. A rat plasma metabolomics based on ultra-performance liquid chromatography coupled with quadrupole time-of-flight mass spectrometry (UPLC-Q-TOF/MS) demonstrated that Tongmai Yangxin pills (TMYXPs) mitigate DOX toxicity by restoring tricarboxylic acid (TCA) cycle activity and modulating key signaling pathways, including insulin, MAPK, and p53, to enhance myocardial protection (Shu et al., 2023). Another study employing 1H-NMR metabolomics highlighted the role of the GPR91 receptor, showing that its agonists (succinate and cis-epoxysuccinate) reduce oxidative stress and apoptosis in DOX-treated cardiomyoblasts by stimulating aerobic metabolism and redox balance in H9C2 cells (Dallons et al., 2020). Another rat plasma metabolomics, screened, validated, and optimized early predictive biomarkers for cardiotoxicity. l-Carnitine, 19-hydroxydeoxycorticosterone, LPC (14:0), and LPC (20:2) exhibited the strongest specificities (Li et al., 2015). Previous metabolomics studies on DOX cardiotoxicity primarily identified dysregulated metabolites (e.g., TCA intermediates, ceramides) as biomarkers but lacked functional validation or mechanistic insights into their roles in cardiac pathology. This superficial association limits clinical translation, as causality between metabolite changes and disease progression remains unproven.

This study aimed to identify potential biomarkers and therapeutic targets for DOX-induced cardiotoxicity by leveraging metabolomics analysis. Using liquid chromatography-mass spectrometry (LC-MS), heart tissue samples from DOX-treated cardiotoxic mice and normal controls were analyzed. The research identified several key metabolites that could serve as reliable biomarkers for early diagnosis. Additionally, we investigated the biological functions of these metabolites, discovering functional metabolites with cardioprotective effects. This work provides valuable insights into mitigating DOX-induced cardiac injury and improving clinical outcomes for patients undergoing chemotherapy.

## Methods

### Establishment of a DOX-Induced cardiotoxicity animal model

Eight-week-old male C57BL/6J mice are randomly assigned to two groups: a control group and an experimental group. The experimental group receives a weekly intraperitoneal injection of DOX at a dose of 5 mg/kg for four consecutive weeks, while the control group is given an equal volume of saline. The body weight of mice in both groups is recorded weekly. In the fifth week, cardiac function is compared between the two groups using M-mode echocardiography, with key parameters including left ventricular ejection fraction (LVEF), left ventricular fractional shortening (LVFS), left ventricular internal diameter at diastole (LVIDd), left ventricular internal diameter at systole (LVIDs), left ventricular posterior wall thickness at diastole (LVPWd) and left ventricular posterior wall thickness at systole (LVPWs). Heart tissues are collected for pathological examination, including Hematoxylin and Eosin (HE) staining, Masson staining and TUNEL staining, to detect cardiac pathological changes. The study procedure was approved by the Ethics Committee of Tongren Hospital, Shanghai Jiao Tong University School of Medicine (approval number A2023-027-01).

### Hematoxylin-eosin (HE) staining of mouse cardiac tissue

Mouse heart tissues were fixed in 4% paraformaldehyde, followed by dehydration through an ascending ethanol gradient (low to high concentration), immersion in absolute ethanol I and II, paraffin embedding, and solidification. Sections (4-5 μm thick) were deparaffinized and rehydrated using a descending ethanol gradient (high to low concentration). The sections were stained with hematoxylin for 3–5 min, differentiated with acid alcohol, counterstained with eosin, and dehydrated again via an ascending ethanol series. Finally, the slides were cleared with xylene, mounted with neutral balsam, and examined under a light microscope for histopathological analysis.

### TUNEL staining for apoptosis detection in mouse cardiac tissue

Apoptosis in cardiac tissue sections was assessed using a TUNEL (Terminal deoxynucleotidyl transferase dUTP nick end labeling) apoptosis detection kit, following the manufacturer’s protocol. Briefly, tissue sections were permeabilized, incubated with TUNEL reaction mixture, and counterstained with DAPI to visualize nuclei. Apoptotic cells exhibited red fluorescence (TUNEL-positive), while normal nuclei appeared blue (DAPI).

### Masson’s trichrome staining of mouse cardiac tissue

Cardiac tissue sections were stained with a Masson’s trichrome kit to evaluate fibrosis. Sections were sequentially immersed in a mixed solution of Masson A and B reagents, differentiated with 1% hydrochloric acid-alcohol, stained with ponceau-acid fuchsin, treated with phosphomolybdic acid solution for 1 min, and then counterstained with aniline blue. After differentiation with 1% acetic acid, sections were dehydrated in absolute ethanol, cleared with xylene, and mounted with neutral balsam. Collagen fibers appeared blue, muscle fibers red, and nuclei dark brown under light microscopy. Fibrotic areas were quantified using image analysis software (Image-Pro Plus) to determine the collagen volume fraction.

### Sample collection and preparation for metabolomic analysis

To prepare cardiac tissue samples for metabolomic analysis, heart tissues are rapidly collected post-mortem, gently washed with physiological saline or ice-cold PBS to remove blood and impurities, and accurately weighed. The samples were rapidly frozen in liquid nitrogen and maintained at −80°C for subsequent processing. Cardiac tissues were homogenized using a Tissuelyser LT (Qiagen) at 30 Hz for 2 min and homogenized in a pre-cooled extraction solvent (80% methanol/water mixture) at a ratio of 1:10 (w/v). The homogenate is incubated at −20°C for 30 min to precipitate proteins, followed by centrifugation at 12, 000 rpm for 15 min at 4°C to collect the supernatant containing metabolites. The supernatant is then dried under vacuum or nitrogen gas. The dried samples are reconstituted in an appropriate solvent (50% methanol/water mixture, 4-chlorophenylalanine (2 μM) as internal standards), filtered through a 0. 22 μm filter to remove particulates, and transferred to autosampler vials for analysis. Throughout the entire process, low temperatures are maintained to minimize metabolite degradation, and precautions are taken to prevent oxidation and cross-contamination. Quality control samples are prepared by pooling equal aliquots from each sample to assess instrument stability and data reproducibility.

### LC/MS analysis

For LC/MS analysis, a Thermo Orbitrap Elite mass spectrometer was paired with an Ultimate 3000 UHPLC system. Separation was carried out using a Kinetex C18 column (100 mm × 2. 1 mm, 1.9 µm particle size). The mobile phase comprised 0.1% formic acid in water (Phase A) and acetonitrile containing 0. 1% formic acid (Phase B), with a gradient program as follows: initial conditions of 98% A and 2% B held for 1 min, followed by a linear increase to 98% B over 8 min (1–9 min), maintained until 12 min. The gradient returned to 98% A at 12.1 min and equilibrated until 15 min. The column temperature was maintained at 25°C, with a flow rate of 0.4 mL/min. A sample volume of 3 µL was injected, and a post-run equilibration time of 5 min was implemented.

Mass spectrometric data were acquired in both positive and negative ionization modes using a Full MS-ddMS2 scan strategy. The scan range covered m/z 100–1,500, with MS1 and MS2 resolutions set to 70,000 and 17,500, respectively. Data-dependent acquisition (TopN = 5) triggered MS2 fragmentation using stepped normalized collision energy (NCE: 30, 40, 50 eV) under higher-energy collisional dissociation (HCD). The automatic gain control (AGC) target was 1e6, and dynamic exclusion was applied for 10 s to avoid redundant fragmentation. For positive ion mode, the ion source parameters included a temperature of 320°C, sheath gas flow of 35 arb, auxiliary gas flow of 15 arb, spray voltage of 3.8 kV, capillary temperature of 350°C, and S-Lens RF level at 30%. Negative ion mode used identical parameters except for a spray voltage of 3.2 kV and S-Lens RF level at 60%.

Raw data were processed using Compound Discoverer 3.3 with a customized metabolomics workflow. Metabolite annotation was performed by matching MS1 and MS2 spectra against the Human Metabolome Database (HMDB), mzCloud (high-resolution MS/MS spectral library), and ChemSpider (chemical structure database). Strict criteria were applied: mass tolerance <5 ppm, retention time deviation <1 min, and a minimum peak intensity threshold of 1,000. Data normalization utilized internal standards and total ion current (TIC) to correct batch effects. A total of 1,500 metabolic features were detected across both ionization modes, with 450 metabolites annotated via HMDB. Differential metabolites (n = 291) were selected based on statistical significance (P < 0.05, VIP > 1) and validated through MS/MS spectral matching (mzCloud score >70%) and pathway enrichment analysis (KEGG, MetaboAnalyst).

### Statistical analysis

To ensure robust handling of technical variability, we implemented a rigorous metabolomics workflow. Batch effects and instrument drift were mitigated through pooled quality control (QC) samples analyzed every 10 injections, enabling retention time alignment via dynamic time warping (DTW) and LOESS regression-based signal correction in Compound Discoverer 3.3. Internal standards (4-chlorophenylalanine, 2 μM) and TIC normalization were applied to minimize inter-batch variability and signal drift. Daily mass spectrometer calibration using Thermo Fisher calibration solutions ensured <2 ppm mass accuracy. Metabolite quantification utilized a 5 ppm mass tolerance and 1 min retention time window, with absolute quantification via internal standards for relative quantification.

Metabolomic data were analyzed utilizing principal component analysis (PCA) and orthogonal partial least squares discriminant analysis (OPLS-DA) through Simca-P software (v11. 0, Umetrics, Sweden). Model validation was achieved by conducting OPLS-DA permutation tests with 200 iterations in both positive and negative ionization modes. Metabolites selected for further investigation were those with a variable importance in projection (VIP) value exceeding 1 and a *p*-value less than 0. 05. Pathway enrichment was performed using MetaboAnalyst 5.0 with KEGG and HMDB annotations. Pathways with a Holm-adjusted p-value <0.05 and impact score >0.1 were considered significant. Comparative analysis of data among different experimental groups were performed using t-tests, with a *p-*value of less than 0.05 considered statistically significant. *p*-values were adjusted using the Benjamini-Hochberg false discovery rate (FDR) method. Additionally, random forest models and receiver operating characteristic (ROC) curves were generated with GraphPad Prism 8 (v8. 0. 1, GraphPad Software, United States).

### Functional validation of key metabolite

In this study, H9C2 rat cardiomyoblast cells were cultured to investigate the protective effects of metabolites D1:N-Stearoylsphingosine, D2: Cer(d18:0/12:0) and D3: 2-Methylglutaric acid against DOX-induced cytotoxicity. The cells were divided into groups: a control group, a DOX group treated with 1 μM DOX to induce cardiotoxicity, and groups pretreated with D1, D2, or D3 at concentrations of 10 μM, 20 μM, or 50 μM for 2 h before DOX exposure. Cell viability was assessed using the CCK-8 assay to determine the ability of the metabolites to mitigate DOX-induced inhibition of cell proliferation.

### Western blot analysis

Western blot analysis was performed to examine the expression levels of apoptosis-related proteins PARP and Caspase-3. Briefly, proteins extracted from treated cells or tissues were separated by SDS-PAGE and transferred onto PVDF membranes. The membranes were blocked with 5% non-fat milk and incubated overnight at 4°C with primary antibodies against PARP (Cell Signaling Technology, #9542, dilution 1:1000), Cleaved Caspase-3 (Cell Signaling Technology, #9661, dilution 1:1000), and GAPDH (Cell Signaling Technology, #5174, dilution 1:1000) as a loading control. After washing, membranes were incubated with HRP-conjugated secondary antibodies (1:5000) for 1 h at room temperature. Protein bands were visualized using an enhanced chemiluminescence (ECL) kit and quantified by densitometry using ImageJ software.

### Mitochondrial membrane potential (MMP) assessment via JC-1 staining

Mitochondrial membrane potential was evaluated using the JC-1 assay kit (cat. no. C2006, Beyotime Institute of Biotechnology, Shanghai, China) following the manufacturer’s instructions. H9C2 cells were seeded into 6-well plates at a density of 2 × 10^5^ cells/well and cultured until reaching 70%–80% confluence. After 24 h of drug treatment, cells were incubated with JC-1 working solution for 10 min at 37°C in the dark. Subsequently, cells were washed three times with serum-free medium and immediately analyzed under a laser scanning confocal microscope (Leica SP8 SR). In healthy mitochondria, JC-1 aggregates emit red fluorescence (excitation/emission: 585/590 nm), whereas depolarized mitochondria show green fluorescence (excitation/emission: 514/529 nm).

## Results

### Construction of a DOX-induced cardiotoxicity model in mice

**FIGURE 1:**
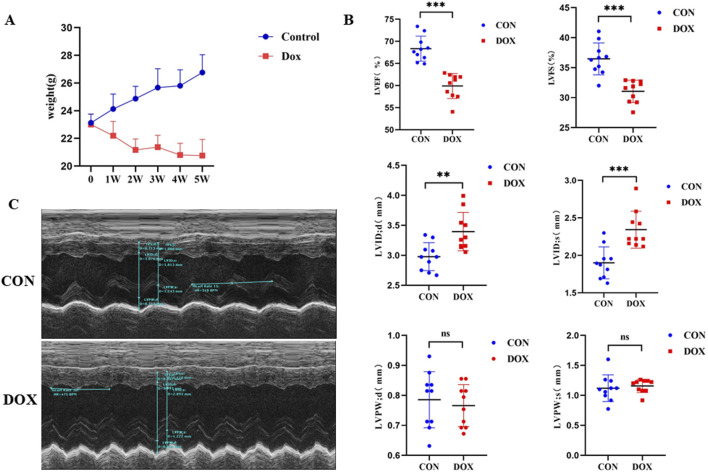
**(A)** Comparison of body weight changes between the two groups of mice during the modeling period; **(B)** Comparison of cardiac ultrasound parameters between the two groups. LVEF (%) is the left ventricular ejection fraction, LVFS (%) is the left ventricular fractional shortening, LVIDd (mm) is the left ventricular internal diameter at diastole. LVIDs (mm) is the left ventricular internal diameter at systole; LVPWd (mm) is the left ventricular posterior wall thickness at diastole; LVPWs (mm) is the left ventricular posterior wall thickness at systol. **(C)** Representative M-mode echocardiographic images of the two groups.

The body weight of mice in the DOX group decreased significantly compared to that of the normal group, indicating that DOX treatment had a significant impact on the overall growth of the mice (Figure 1A). However, cardiac ultrasound results revealed that, compared to the control group, the model group exhibited a significant decrease in LVEF and LVFS (P < 0. 05), suggesting impaired left ventricular systolic function. LVIDd and LVIDs was significantly increased (P < 0. 05). This indicates changes in systolic and diastolic function of the heart (Figure 1B). No significant differences were observed between the DOX and control group in parameters such as LVPWd and LVPWs (Figure 1B).

Furthermore, representative M-mode echocardiographic images demonstrated normal cardiac structure and function in control mice, while mice in the model group showed increased left ventricular diameter, reduced wall thickness, and weakened systolic function, consistent with the quantitative ultrasound parameters (Figure 1C). In summary, DOX treatment led to significant changes in cardiac function in mice, mainly manifested as decreased left ventricular systolic function and structural alterations, providing experimental evidence for further exploration of its mechanism of action.

### Histological assessment of myocardial tissue

**FIGURE 2:**
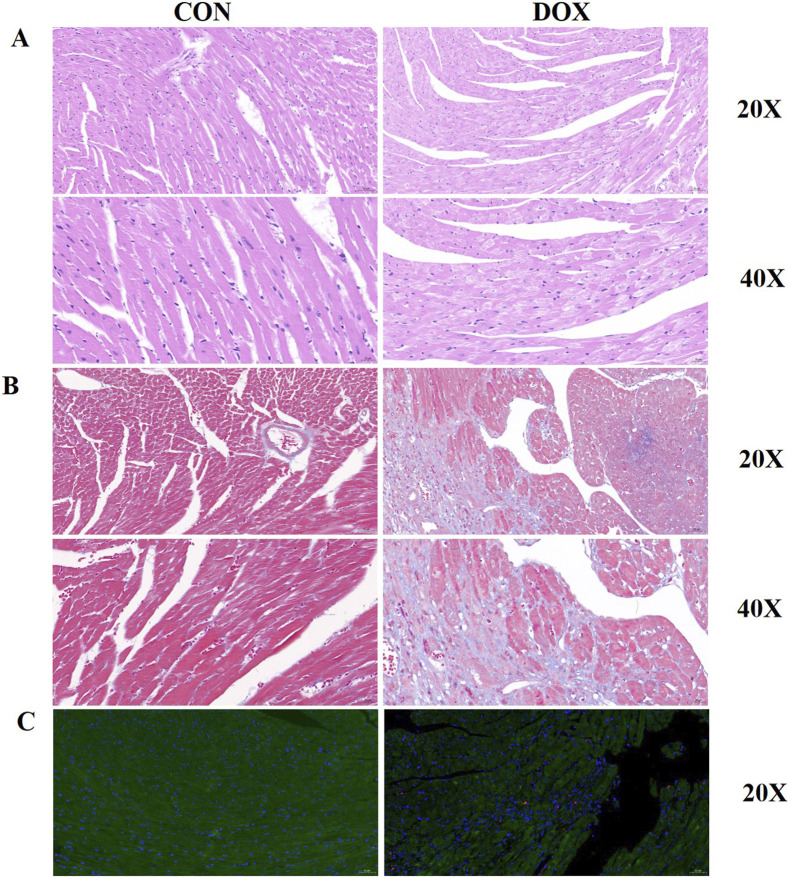
**(A)** HE staining of myocardial tissue from control and DOX-treated groups. The DOX group exhibits disordered cell arrangement, widened muscle fiber gaps, and vacuole-like changes in the cytoplasm; **(B)** Masson’s trichrome staining of myocardial tissue from control and DOX-treated groups. The DOX group displays pronounced myocardial fibrosis with increased collagen fiber accumulation; **(C)** TUNEL staining of myocardial tissue from control and DOX-treated groups. The DOX group shows a significant increase in apoptotic myocardial cells.

To further evaluate the extent of myocardial damage induced by DOX in mice, the histological examination of heart tissue was performed using HE staining, Masson’s trichrome staining, and TUNEL staining techniques. The results revealed significant pathological changes in the myocardial tissue of the DOX-treated group compared to the control group. In the HE staining, myocardial fibers in the control group were tightly arranged with clear and intact structures. In contrast, the DOX group exhibited disorganized arrangement of cardiomyocytes, noticeably widened gaps between muscle fibers, pale cytoplasm, and vacuole-like changes, indicating cellular edema and structural disruption (Figure 2A). Masson’s trichrome staining showed minimal collagen fiber deposition in the myocardial tissue of the control group. However, the DOX group displayed significant myocardial fibrosis, with abundant blue-stained collagen fibers accumulating in the myocardial interstitium, indicating a marked increase in fibrosis (Figure 2B). TUNEL staining was used to detect apoptosis in cardiomyocytes. The control group showed few TUNEL-positive cells, indicating a low level of apoptosis. In contrast, the DOX-treated group exhibited a significant increase in TUNEL-positive cells, demonstrating a marked enhancement of cardiomyocyte apoptosis (Figure 2C).

In summary, DOX treatment led to substantial structural damage in the myocardial tissue of mice, including disorganized cell arrangement, increased fibrosis, and elevated apoptosis. These histological changes may be the primary reasons for the DOX-induced impairment of cardiac function.

### Metabolomic profiling of heart tissues

**FIGURE 3:**
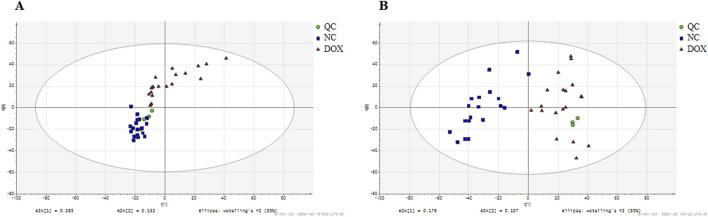
**(A)** PCA plots of the differential metabolites among the groups in positive ion mode. **(B)** PCA plots of the differential metabolites among the groups in negative ion mode.

**FIGURE 4:**
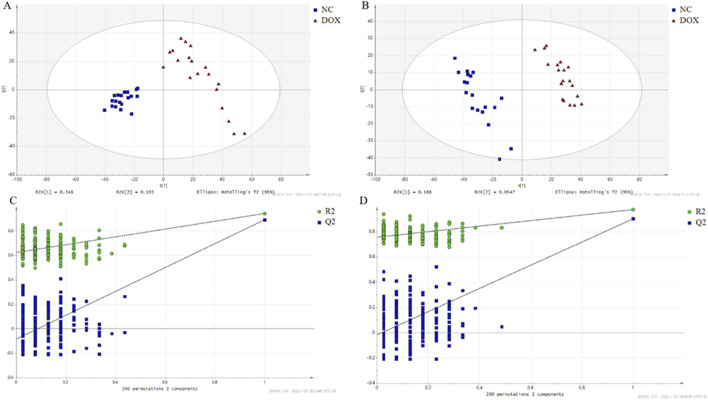
The score plot based on OPLS-DA model. **(A)** OPLS-DA score map in positive ion mode; **(B)** OPLS-DA score map in negative ion mode; **(C)** Permutation test with the OPLS-DA model in positive ion mode; **(D)** Permutation test with OPLS-DA model in negative mode.

Heart tissue analysis was performed using untargeted metabolomics, followed by data processing. To visualize the differences in metabolite profiles between the control group and the DOX-induced cardiotoxicity group, PCA was used (Figure 3). PCA results demonstrated distinct group clustering, with a clear separation observed between the DOX group and the control group, suggesting significant metabolic differences. To further identify the metabolites contributing to these differences, OPLS-DA was performed, and score plots for both groups were generated under positive and negative ionization modes, utilizing 200-fold cross-validation for model validation (Figure 4).

**FIGURE 5:**
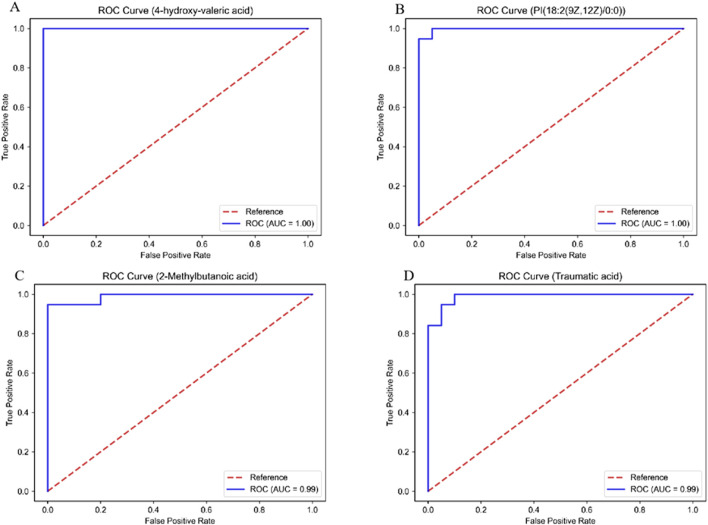
ROC curves of the key differentially abundant metabolites between the DOX group and the CON group. **(A)** 4-hydroxy-valeric acid; **(B)** PI [18:2 (9Z, 12Z)/0:0]; **(C)** 2-methylbutanoic acid; **(D)** traumatic acid.

**FIGURE 6:**
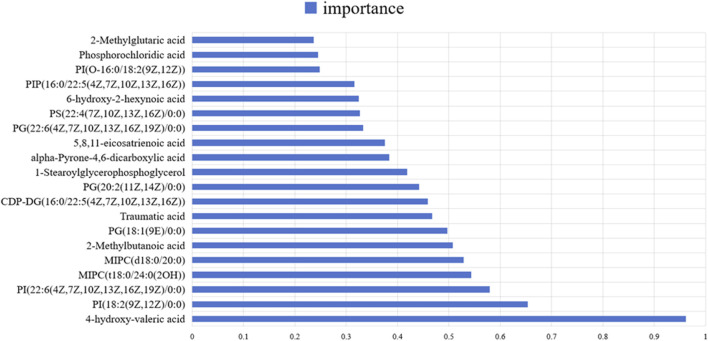
The key differentially abundant metabolites identified by applying the random forest algorithm between the control group and the DOX group.

**FIGURE 7:**
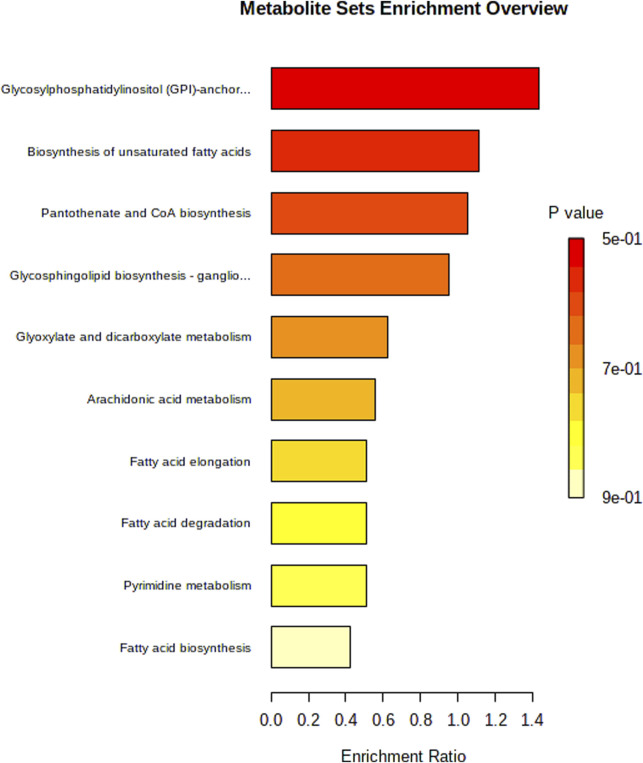
The horizontal bar chart of KEGG pathway analysis.

A total of 291 differential metabolites were identified between the DOX group and the control group (Supplementary Table S1). The volcano plot has been deposited in Supplementary materials as shown in Supplementary Figure S1. Among the differential metabolites, five were identified as having potential diagnostic value for DOX-induced cardiotoxicity: 4-hydroxy-valeric acid, 2-methylbutanoic acid, traumatic acid, PI [18:2 (9Z, 12Z)/0:0], and MIPC [t18:0/24:0 (2OH)]. ROC curve analysis demonstrated excellent diagnostic performance for 4-hydroxy-valeric acid and PI [18:2 (9Z, 12Z)/0:0], both achieving an area under the curve (AUC) of 1. 00. Additionally, 2-methylbutanoic acid showed high discriminatory power with an AUC of 0. 99 (Figure 5). The random forest algorithm was applied to analyze 291 distinct metabolites identified between the DOX group and the Control group. The top 20 significantly differentially expressed metabolites were selected as predictive markers (Figure 6). The pathway enrichment analysis of the altered metabolites identified numerous significant metabolic processes related to DOX-induced cardiotoxicity. Notably, alterations were observed in glycosylphosphatidylinositol-anchor biosynthesis, unsaturated fatty acid biosynthesis, and pathways involving pantothenate and CoA metabolism. These pathways are crucial for cardiac function and energy metabolism, suggesting that DOX disrupts essential metabolic processes in cardiomyocytes (Figure 7).

### Functional validation of key metabolite

**FIGURE 8:**
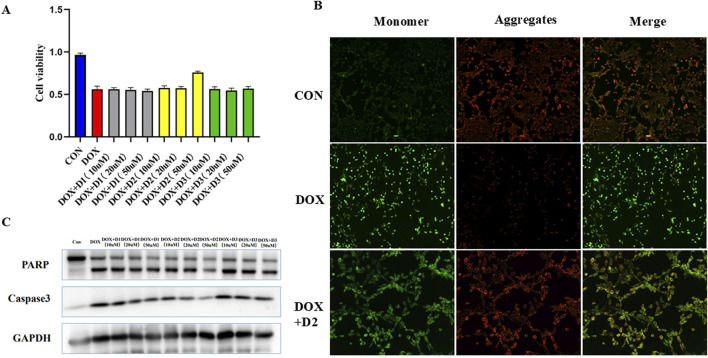
**(A)** Effect of D2 on DOX-induced inhibition of H9C2 cell proliferation. H9C2 cells were treated with DOX (1 μM) in the presence or absence of D2 at concentrations of 10 μM, 20 μM, and 50 μM. D1: N-Stearoylsphingosine D2: Cer (d18:0/12:0) D3:2-Methylglutaric acid **(B)** D2 restores mitochondrial membrane potential in DOX-treated H9C2 cells. Representative fluorescence microscopy images showing JC-1 staining in different treatment groups. Healthy mitochondria exhibit red fluorescence, whereas depolarized mitochondria show green fluorescence. **(C)** Effect of D2 on the expression of apoptosis-related proteins in DOX-treated H9C2 cells. Representative Western blot images showing the expression of PARP, Caspase-3, and GAPDH (loading control).

Further investigation focused on one differential metabolite, Cer (d18:0/12:0), to validate its role in DOX-induced cardiotoxicity. *In vitro* experiments demonstrated that Cer (d18:0/12:0) could ameliorate DOX-induced damage in myocardial cells. Treatment with this metabolite improved cell viability and inhibited the expression of apoptosis-related proteins, indicating its potential protective effect against DOX-induced apoptosis in cardiomyocytes. The results demonstrated that among the tested metabolites, D2 [Cer (d18:0/12:0)] at a concentration of 50 μM significantly alleviated the inhibitory effect of DOX on H9C2 cell proliferation, as indicated by increased cell viability compared to the DOX-only group (Figure 8A). Additionally, D2 improved the mitochondrial membrane potential in DOX-treated cells, evidenced by an increased red/green fluorescence ratio in the mitochondrial membrane potential JC-1 assay, suggesting protective effects on mitochondrial function (Figure 8B). Western blot analysis revealed that 50 μM D2 downregulated the expression of apoptosis-related proteins PARP and Caspase-3 in H9C2 cells exposed to DOX, indicating that D2 can inhibit DOX-induced apoptosis (Figure 8C). These findings suggest that D2 has a protective role against DOX-induced cardiotoxicity by improving cell viability, preserving mitochondrial function, and reducing apoptosis in H9C2 cells.

## Discussion

The chemotherapeutic agent DOX is a highly effective and widely used antitumor drug in the clinical treatment of various malignancies. However, its dose-dependent cardiotoxicity can lead to irreversible myocardial damage, potentially resulting in congestive heart failure. This severe side effect limits the clinical application of DOX and adversely affects patient quality of life and prognosis. Therefore, there is significant clinical importance and urgency in deeply investigating the mechanisms underlying DOX-induced cardiotoxicity, identifying reliable predictive biomarkers, and developing effective cardioprotective agents.

In this study, we utilized metabolomic analysis and cellular experiments to explore DOX-induced cardiotoxicity and potential biomarkers, focusing particularly on the impact of the differential metabolite Cer (d18:0/12:0) on cardiomyocyte apoptosis.

Our metabolomic analysis revealed significant alterations in the metabolic profiles of cardiac tissues in the DOX-induced cardiotoxicity model. A total of 291 metabolites were detected, with several showing significant differences between the model group and the control group. PCA and OPLS-DA clarified the metabolic differences between the two groups. These differential metabolites mainly involved key pathways such as energy metabolism, lipid metabolism, and oxidative stress.

Notably, metabolites like 4-hydroxy-valeric acid, 2-methylbutanoic acid, traumatic acid, PI [18:2 (9Z, 12Z)/0:0], and MIPC [t18:0/24:0 (2OH)] were identified as potential diagnostic biomarkers. ROC curve analysis showed that 4-hydroxy-valeric acid and PI [18:2 (9Z, 12Z)/0:0] had area under the curve (AUC) values of 1. 00, and 2-methylbutanoic acid had an AUC of 0. 99, indicating extremely high discriminative ability. The significant changes in these metabolites suggest that they may play crucial roles in DOX-induced cardiotoxicity.

4-Hydroxy-valeric acid has emerged as a compound of interest in cardiotoxicity research due to its potential roles in cardiac health. DOX induce cardiotoxicity through mechanisms such as endothelial dysfunction, which drives cardiomyopathy and heart failure (Grakova et al., 2021). 4-Hydroxy-valeric acid may mitigate these effects by influencing endothelial integrity or modulating cellular stress pathways, including oxidative and inflammatory responses-key targets for cardiovascular risk reduction (Mooradian, 2017). Additionally, insights from drug-lipid interaction studies, particularly in NSAID-induced cardiotoxicity, suggest that 4-hydroxy-valeric acid could alter membrane dynamics to protect cardiac cells (Pereira-Leite et al., 2020). These multifaceted roles-spanning endothelial function, stress regulation, and lipid interactions-highlight its therapeutic potential and underscore the need for mechanistic studies to validate its cardioprotective effects.

2-Methylbutanoic acid, a short-chain fatty acid (SCFA), shows potential cardioprotective roles by modulating oxidative stress and mitochondrial dysfunction-mechanisms highlighted in studies of structurally related compounds. For instance, phenylalanine-butyramide, a butyrate derivative, protects against DOX-induced cardiotoxicity by reducing oxidative stress, improving mitochondrial function, and preventing left ventricular remodeling (Russo et al., 2019). Similarly, 2-methylbutanoic acid may act through SCFA-mediated pathways to counteract metabolic imbalances in cardiac disease states, where altered fatty acid metabolism critically impacts energy homeostasis and pathology progression (Stamenkovic et al., 2019). Additionally, research on environmental pollutants like tributyltin underscores the broader relevance of lipid-mediated oxidative stress and calcium dysregulation in cardiotoxicity (Pereira et al., 2019), suggesting that 2-methylbutanoic acid’s effects on these pathways warrant further mechanistic exploration.

Traumatic acid, has emerged as a potential cardioprotective agent in drug-induced cardiotoxicity models, particularly for its antioxidative and anti-inflammatory properties. DOX-induce cardiotoxicity via oxidative stress pathways, driving cardiomyocyte damage and heart failure (Fabiani et al., 2021). Traumatic acid may counteract these effects by modulating redox balance, akin to phenolic acids like rosmarinic acid, which mitigate drug-induced cardiac injury (e.g., arsenic trioxide) through similar mechanisms (Liu et al., 2022). Additionally, its role in oxidative stress regulation aligns with efforts to identify biomarkers (e.g., lipid peroxidation products) for early detection of anthracycline cardiotoxicity (Dickey and Rao, 2012). These dual therapeutic and diagnostic potentials position traumatic acid as a promising candidate for integrative strategies to enhance cardiac safety in chemotherapy.

Phosphatidylinositol (PI) species, such as PI (18:2/0:0), play critical roles in various cellular processes, including signal transduction and membrane dynamics. In the context of cardiotoxicity models, these lipid molecules are particularly significant due to their involvement in the regulation of cardiac health and disease mechanisms. One study highlights the importance of lipid metabolism in cardiac function, particularly in the context of cardiac arrest models. It was observed that lipid metabolism, including specific PI species, can influence myocardial function and recovery post-resuscitation. The study suggests that lipidomic profiling, including PI species, could serve as potential biomarkers for cardiac outcomes and therapeutic targets to improve cardiac health following cardiac arrest (Xiao et al., 2020). Additionally, distinct lipidomic profiles have been identified in models of physiological and pathological cardiac remodeling, suggesting that specific lipid species, including PIs, are differentially regulated in these conditions. This differential regulation could potentially be leveraged to modulate heart size and function, offering a therapeutic avenue for addressing cardiac remodeling and dysfunction (Tham et al., 2018).

MIPC [t18:0/24:0 (2OH)], a complex sphingolipid, is critical for cardiac health through its roles in membrane integrity, signal transduction, and cellular homeostasis. Sphingolipid metabolism influences cardiac stress responses, as evidenced by studies showing enhanced sphingolipid synthesis improves osmotic tolerance in yeast—a protective mechanism potentially translatable to stressed cardiomyocytes (Zhu et al., 2020). In cardiac diseases, sphingolipid dysregulation disrupts lipid metabolism and energy homeostasis, contributing to systemic metabolic dysfunction and cardiovascular pathologies (Liu et al., 2013). Furthermore, sphingolipids like MIPC modulate oxidative stress and mitochondrial dynamics, key drivers of cardiomyocyte apoptosis during cardiotoxicity (Aung et al., 2019). These roles position MIPC [t18:0/24:0 (2OH)] as a potential biomarker and therapeutic target for mitigating cardiac damage and restoring metabolic balance.

Pathway analysis further revealed that these differential metabolites are involved in biological processes such as glycosylphosphatidylinositol-anchor biosynthesis, unsaturated fatty acid biosynthesis, and pantothenate and CoA metabolism. These pathways are closely related to cardiac energy supply, membrane structural integrity, and cellular signal transduction. DOX may cause myocardial cell dysfunction and increased apoptosis by interfering with these critical metabolic pathways.

One of the primary mechanisms by which DOX induces cardiotoxicity is through oxidative stress. DOX is known to undergo redox cycling, leading to the generation of reactive oxygen species (ROS) within cardiomyocytes. This increase in ROS results in oxidative damage to cellular components, including lipids, proteins, and DNA, ultimately leading to cell death and cardiomyopathy. The oxidative stress pathway is a significant contributor to the cardiotoxic effects of DOX, as it disrupts the balance between pro-oxidants and antioxidants, leading to cellular damage and apoptosis (Kalyanaraman, 2020; Avagimyan et al., 2024).

Lipid metabolism is another critical pathway affected by DOX treatment. DOX-induced cardiotoxicity involves alterations in mitochondrial lipid metabolism, which can lead to bioenergetic failure and cell death. The disruption of lipid metabolism is characterized by impaired fatty acid oxidation and an accumulation of lipids within the myocardium. This lipid overload can exacerbate oxidative stress and contribute to mitochondrial dysfunction, further promoting cardiomyocyte apoptosis and cardiac dysfunction (Carvalho et al., 2014; Casquel et al., 2018). CoA metabolism is also implicated in DOX-induced cardiotoxicity. CoA is essential for various metabolic pathways, including the synthesis and oxidation of fatty acids. Alterations in CoA metabolism can disrupt these processes, leading to an accumulation of toxic lipid intermediates and further exacerbating oxidative stress. The interplay between CoA metabolism and lipid metabolism is crucial in understanding the metabolic derangements that occur in DOX-induced cardiotoxicity (Ni et al., 2019; Arunachalam et al., 2021).

In summary, DOX-induced cardiotoxicity is a complex process involving multiple altered pathways. Oxidative stress plays a central role by damaging cellular components and promoting apoptosis. Alterations in lipid metabolism contribute to mitochondrial dysfunction and bioenergetic failure, while disruptions in CoA metabolism exacerbate these effects. Understanding these pathways provides insights into potential therapeutic strategies to mitigate the cardiotoxic effects of DOX (Arunachalam et al., 2021; Hu et al., 2020).

Our metabolomic analysis offers valuable insights for identifying specific biomarkers of DOX-induced cardiotoxicity. The differential metabolites and enriched metabolic pathways uncovered enhance our understanding of the mechanisms behind DOX cardiotoxicity. Notably, metabolites with high diagnostic potential, such as 4-hydroxy-valeric acid and PI [18:2 (9Z, 12Z)/0:0], could serve as specific biomarkers for predicting and monitoring cardiotoxicity caused by chemotherapy drugs. Among the identified differential metabolites, Cer(d18:0/12:0) attracted our attention. Ceramides are important sphingolipid metabolites involved in regulating processes such as cell proliferation, differentiation, and apoptosis. Our cellular experiments demonstrated that Cer (d18:0/12:0) at a concentration of 50 μM significantly alleviated the inhibitory effect of DOX on the proliferation of H9C2 cardiomyocytes, increasing cell viability. Additionally, Cer (d18:0/12:0) improved the DOX-induced decrease in mitochondrial membrane potential, indicating a protective effect on mitochondrial function.

Further Western blot analysis showed that Cer (d18:0/12:0) downregulated the expression levels of apoptosis-related proteins PARP and Caspase-3 induced by DOX, inhibiting the apoptosis process. These results collectively suggest that Cer (d18:0/12:0) protects cardiomyocytes from DOX-induced toxic damage, potentially through inhibiting the mitochondrial-mediated apoptosis pathway.

In addition, we are concerned about previous study reporting that elevated ceramide levels are associated with adverse cardiovascular risks and events (Shu et al., 2022). Another study showed that different types of ceramides contribute differently to the risk of heart failure, higher plasma Cer-16 levels were associated with an increased risk of heart failure, while Cer-22 levels were associated with a reduced risk of heart failure (Lemaitre et al., 2019). Ceramide (C16, C20, C20:1, and C24) levels increased significantly 24 h after myocardial infarction, and the role of C18 was not mentioned (Hadas et al., 2020). Fecal metabolites Cer (d18:0/12:0) and Cer (d18:0/14:0) are reduced in patients with RA, which may be protective factor for RA (Yu et al., 2022). Therefore, more research is needed to further identify and distinguish the functions of different types of ceramides, revealing their underlying mechanisms.

In conclusion, the combination of metabolomics and machine learning enabled the identification of five metabolites that could act as promising biomarkers for diagnosing DOX-induced cardiotoxicity. Beyond serving as biomarkers, we also found that Cer (d18:0/12:0) has the potential to protect the heart. Since it can inhibit cardiomyocyte apoptosis, future studies can further investigate its mechanism of action and evaluate its cardioprotective effects *in vivo.* If its safety and efficacy can be demonstrated, Cer (d18:0/12:0) may be developed into a therapeutic agent to prevent or mitigate cardiotoxicity from chemotherapy drugs, offering new strategies for cardiac protection in cancer patients.

The clinical translation of Cer (d18:0/12:0) faces multifaceted challenges, including species-specific metabolic disparities, pre-analytical variability in metabolite stability, and patient heterogeneity in cardiotoxicity susceptibility. Additionally, achieving cardiac-targeted delivery while minimizing off-target effects and addressing high development costs requires advanced formulation optimization and strategic clinical trial designs. Overcoming these barriers demands interdisciplinary collaboration and robust preclinical-to-clinical validation frameworks.

## Conclusion

In summary, this study identified potential biomarkers of DOX-induced cardiotoxicity through metabolomic analysis and discovered the differential metabolite Cer(d18:0/12:0) that has cardioprotective effects. These findings provide an important foundation for understanding the mechanisms of DOX cardiotoxicity and developing new diagnostic and therapeutic approaches, with significant clinical application prospects.

## Data Availability

The datasets presented in this study can be found in online repositories. The names of the repository/repositories and accession number(s) can be found in the article/Supplementary Material.
