# Supplementary material for: Metabolomic profiling and biomarker identification for early detection and therapeutic targeting of doxorubicin-induced cardiotoxicity
Source: Front Cell Dev Biol. 2025 Apr 10;13:1543636. doi: 10.3389/fcell.2025.1543636 (PMC12018317; doi:10.3389/fcell.2025.1543636)
Supplement: Supplementary file 1 [file DataSheet1.docx]

**Metabolomic Profiling and Biomarker Identification for Early Detection and Therapeutic Targeting of Doxorubicin-Induced Cardiotoxicity**

Jingjing Ding^△1,2^, Xianzhen Feng^△2^, Zhongqing Xu^*2^, Hong Xu^*1^

1. Department of Oncology, The First Affiliated Hospital of Soochow University, Suzhou, China.

2. Department of General Practice, Tongren Hospital, Shanghai Jiao Tong University School of Medicine, Shanghai, China.

△ co-first authors

* corresponding author

Table S1. Differentially abundant metabolites between the Control group and the DOX group.

| VIP | Name | HMDB_ID | m/z | RT(min) | log2fc(DOX/NC） | p |
| --- | --- | --- | --- | --- | --- | --- |
| 1.09 | TG(18:0/20:3(5Z,8Z,11Z)/O-18:0) | HMDB0045018 | 897.8114 | 10.07 | #DIV/0! | 7.52E-03 |
| 1.02 | Ceramide (d18:1/20:0) | HMDB0004951 | 592.5675 | 10.03 | 8.57 | 8.17E-03 |
| 1.09 | TG(18:0/22:5(4Z,7Z,10Z,13Z,16Z)/O-18:0) | HMDB0045288 | 921.8126 | 9.95 | 5.52 | 7.43E-03 |
| 1.01 | PC(O-14:0/15:0) |  | 678.5459 | 7.57 | 4.90 | 2.02E-02 |
| 1.24 | PE(14:0/P-18:0) | HMDB0008851 | 676.5313 | 7.66 | 3.13 | 6.43E-04 |
| 1.11 | PI(20:1(11Z)/22:2(13Z,16Z)) |  | 945.6696 | 9.10 | 2.68 | 1.58E-03 |
| 1.59 | CL(16:0/16:0/16:0/16:1(9Z)) | HMDB0056389 | 1349.9247 | 9.31 | 2.39 | 2.69E-06 |
| 1.36 | PA(O-20:0/16:0) |  | 691.5611 | 7.58 | 2.02 | 1.48E-04 |
| 1.26 | PA(O-16:0/20:2(11Z,14Z)) |  | 685.5148 | 10.82 | 1.78 | 7.98E-04 |
| 1.29 | PE-NMe(16:0/24:0) | HMDB0113059 | 816.6560 | 8.68 | 1.76 | 6.38E-04 |
| 1.48 | MIPC(d18:0/16:0) |  | 944.6031 | 8.86 | 1.66 | 1.05E-06 |
| 1.31 | Homophytanic acid | HMDB0002337 | 327.3301 | 8.03 | 1.64 | 6.24E-04 |
| 1.05 | PIP(18:0/20:3(5Z,8Z,11Z)) | HMDB0009954 | 1043.5941 | 8.22 | 1.46 | 2.29E-02 |
| 1.31 | GlcCer(d14:2(4E,6E)/18:1(9Z)) |  | 668.4986 | 7.72 | 1.44 | 1.23E-04 |
| 1.72 | 5-Carboxy-2'-deoxyuridine | HMDB0060774 | 271.0573 | 1.04 | 1.40 | 1.78E-07 |
| 1.26 | Oleoyl Ethyl Amide |  | 310.3041 | 11.17 | 1.34 | 1.83E-04 |
| 1.64 | LysoPC(15:0/0:0) | HMDB0010381 | 480.3096 | 9.25 | 1.29 | 1.12E-06 |
| 1.13 | Perfluorotetracosane |  | 1238.8912 | 8.70 | 1.27 | 9.96E-04 |
| 1.05 | PG(O-16:0/15:1(9Z)) |  | 693.5152 | 7.63 | 1.26 | 2.48E-02 |
| 1.39 | PI(13:0/22:2(13Z,16Z)) |  | 849.5434 | 11.81 | 1.23 | 1.52E-05 |
| 1.54 | Platydesminium | HMDB0030342 | 275.1519 | 0.96 | 1.15 | 2.62E-07 |
| 1.35 | PS(19:1(9Z)/22:6(4Z,7Z,10Z,13Z,16Z,19Z)) |  | 848.5399 | 11.81 | 1.03 | 3.30E-05 |
| 1.12 | PI(22:0/22:4(7Z,10Z,13Z,16Z)) |  | 971.6627 | 8.86 | 0.98 | 6.13E-04 |
| 1.27 | TG(24:0/20:3(5Z,8Z,11Z)/O-18:0) | HMDB0047316 | 981.9497 | 0.62 | 0.98 | 5.38E-04 |
| 1.28 | TG(19:0/22:0/22:0)[iso3] |  | 1015.9585 | 0.62 | 0.97 | 5.04E-04 |
| 1.80 | PE-NMe2(22:6(4Z,7Z,10Z,13Z,16Z,19Z)/22:6(4Z,7Z,10Z,13Z,16Z,19Z)) | HMDB0114601 | 864.5537 | 10.71 | 0.96 | 2.48E-11 |
| 1.49 | CL(8:0/8:0/11:0/18:2(9Z,11Z)) | HMDB0116884 | 1083.6601 | 8.87 | 0.96 | 7.97E-07 |
| 1.42 | CDP-DG(a-15:0/a-25:0) | HMDB0116085 | 1064.6051 | 8.24 | 0.95 | 6.66E-05 |
| 1.63 | Ganglioside GA2 (d18:1/12:0) | HMDB0004888 | 1009.6118 | 8.58 | 0.93 | 1.61E-08 |
| 1.11 | TG(16:0/16:1(9Z)/16:1(9Z)) | HMDB0005376 | 801.7035 | 8.68 | 0.88 | 2.54E-03 |
| 1.15 | Glucosylceramide (d18:1/9Z-18:1) | HMDB0004970 | 724.5829 | 8.68 | 0.87 | 1.82E-03 |
| 1.13 | Ganglioside GA1 (d18:1/20:0) | HMDB0004904 | 1281.8426 | 8.15 | 0.82 | 2.07E-03 |
| 1.03 | Ganglioside GM3 (d18:1/26:1(17Z))) | HMDB0004849 | 1291.8337 | 8.15 | 0.77 | 1.87E-03 |
| 1.05 | PE-NMe(15:0/22:6(4Z,7Z,10Z,13Z,16Z,19Z)) | HMDB0113036 | 762.5090 | 10.21 | 0.76 | 6.83E-03 |
| 1.22 | Helianyl octanoate | HMDB0038337 | 555.5102 | 6.73 | 0.75 | 1.49E-04 |
| 1.13 | PS(20:4(5Z,8Z,11Z,14Z)/22:6(4Z,7Z,10Z,13Z,16Z,19Z)) | HMDB0012439 | 856.4967 | 7.18 | 0.75 | 7.24E-03 |
| 1.46 | alpha-Solanine | HMDB0034202 | 868.5183 | 10.78 | 0.72 | 2.27E-06 |
| 1.05 | Narasin | HMDB0030448 | 763.5121 | 10.20 | 0.72 | 6.35E-03 |
| 1.47 | Hexanal dihexyl acetal | HMDB0032316 | 287.2983 | 10.97 | 0.71 | 1.52E-06 |
| 1.50 | Cer(d18:1/22:1(13Z)) | HMDB0011775 | 620.6061 | 11.08 | 0.71 | 9.34E-07 |
| 1.04 | TG(22:4(7Z,10Z,13Z,16Z)/22:6(4Z,7Z,10Z,13Z,16Z,19Z)/O-18:0) | HMDB0054900 | 967.8297 | 8.68 | 0.71 | 5.19E-03 |
| 1.00 | TG(22:4(7Z,10Z,13Z,16Z)/22:5(4Z,7Z,10Z,13Z,16Z)/O-18:0) | HMDB0054846 | 969.8346 | 8.68 | 0.70 | 7.34E-03 |
| 1.05 | TG(18:3(9Z,12Z,15Z)/20:1(11Z)/20:1(11Z))[iso3] |  | 935.7799 | 8.68 | 0.70 | 4.53E-03 |
| 1.31 | PS(14:0/20:4(8Z,11Z,14Z,17Z)) | HMDB0112298 | 754.4671 | 11.86 | 0.70 | 3.23E-04 |
| 1.41 | Diadenosine heptaphosphate | HMDB0001433 | 1074.9116 | 0.62 | 0.69 | 7.36E-05 |
| 1.71 | C-6 NBD Ceramide |  | 574.3520 | 10.18 | 0.67 | 2.27E-07 |
| 1.02 | PE(20:0/P-18:0) | HMDB0009247 | 758.6036 | 8.68 | 0.66 | 6.02E-03 |
| 1.53 | PI(16:0/20:0) | HMDB0009785 | 867.5813 | 9.21 | 0.63 | 2.93E-07 |
| 1.02 | PC(18:1(11Z)/22:6(4Z,7Z,10Z,13Z,16Z,19Z)) | HMDB0008090 | 832.5708 | 11.80 | 0.63 | 2.79E-03 |
| 1.70 | CDP-DG(16:0/22:5(4Z,7Z,10Z,13Z,16Z)) | HMDB0115950 | 1028.5675 | 7.98 | 0.62 | 1.65E-09 |
| 1.27 | PS(22:1(11Z)/0:0) |  | 578.3463 | 9.80 | 0.60 | 8.46E-04 |
| 1.55 | PS(22:4(7Z,10Z,13Z,16Z)/22:6(4Z,7Z,10Z,13Z,16Z,19Z)) |  | 884.5280 | 10.72 | 0.60 | 1.90E-07 |
| 1.37 | PS(16:1(9Z)/18:1(9Z)) | HMDB0012368 | 758.5001 | 10.22 | 0.60 | 1.37E-04 |
| 1.51 | PS(20:2(11Z,14Z)/22:6(4Z,7Z,10Z,13Z,16Z,19Z)) |  | 860.5303 | 12.17 | 0.59 | 5.09E-07 |
| 1.20 | Uridine diphosphate-N-acetylglucosamine | HMDB0000290 | 606.0740 | 0.94 | 0.59 | 1.36E-03 |
| 1.38 | Dephospho-CoA | HMDB0001373 | 686.1411 | 2.01 | 0.59 | 4.68E-04 |
| 1.75 | PI(21:0/22:6(4Z,7Z,10Z,13Z,16Z,19Z)) |  | 968.6516 | 8.89 | 0.58 | 2.94E-07 |
| 1.03 | PS(16:0/22:4(7Z,10Z,13Z,16Z)) | HMDB0112352 | 812.5438 | 9.91 | 0.58 | 5.93E-03 |
| 1.43 | Cer(d18:0/18:0) | HMDB0011761 | 568.5755 | 10.97 | 0.58 | 2.89E-06 |
| 1.59 | N-palmitoyl glutamine |  | 383.2916 | 8.98 | 0.57 | 1.56E-05 |
| 1.22 | Glycyrrhetinic acid | HMDB0011628 | 471.3498 | 7.00 | 0.57 | 9.46E-04 |
| 1.29 | CL(i-13:0/i-22:0/i-12:0/i-13:0) | HMDB0073652 | 1297.8937 | 8.87 | 0.57 | 2.85E-04 |
| 2.16 | Mivacurium | HMDB0015357 | 1027.5704 | 7.98 | 0.56 | 6.67E-16 |
| 1.17 | PI-Cer(d18:0/16:0) |  | 782.5584 | 9.91 | 0.56 | 2.98E-04 |
| 1.31 | CL(16:0/16:0/16:1(9Z)/20:4(5Z,8Z,11Z,14Z)) | HMDB0056413 | 1397.9238 | 8.14 | 0.56 | 3.06E-04 |
| 1.61 | PA(P-20:0/19:1(9Z)) |  | 729.5785 | 14.00 | 0.56 | 1.62E-06 |
| 1.33 | Melilotussaponin O1 | HMDB0031965 | 1073.5633 | 7.96 | 0.56 | 1.91E-04 |
| 1.10 | PS(18:1(9Z)/22:2(13Z,16Z)) |  | 840.5747 | 8.82 | 0.55 | 4.08E-03 |
| 1.36 | Ganglioside GM3 (d18:1/26:0) | HMDB0004850 | 1291.8286 | 7.99 | 0.55 | 1.74E-04 |
| 1.13 | PG(16:0/0:0) |  | 483.2734 | 10.82 | 0.54 | 2.01E-03 |
| 1.32 | CL(18:2(9Z,12Z)/18:2(9Z,12Z)/18:2(9Z,12Z)/16:1(9Z)) | HMDB0010245 | 1421.9228 | 8.15 | 0.54 | 2.10E-04 |
| 1.20 | Taurocholic acid | HMDB0000036 | 516.2977 | 7.57 | 0.54 | 1.34E-03 |
| 1.50 | Oleyloxyethyl Phosphorylcholine |  | 478.3718 | 7.56 | 0.53 | 3.58E-06 |
| 1.39 | PS(18:3(6Z,9Z,12Z)/22:6(4Z,7Z,10Z,13Z,16Z,19Z)) | HMDB0112465 | 828.5012 | 10.82 | 0.53 | 8.19E-05 |
| 2.03 | Gluten exorphin C | HMDB0059796 | 666.3980 | 9.76 | 0.53 | 2.63E-12 |
| 1.17 | 3,4,5-Tribromobenzene-1,2-dicarboxylic acid |  | 400.7651 | 13.97 | 0.51 | 1.11E-03 |
| 1.23 | Goshonoside F6 | HMDB0038542 | 617.3527 | 8.90 | 0.50 | 1.67E-04 |
| 1.15 | PC(22:5(4Z,7Z,10Z,13Z,16Z)/24:1(15Z)) | HMDB0008684 | 918.7011 | 0.62 | 0.50 | 4.11E-04 |
| 1.31 | PA(O-20:0/19:1(9Z)) |  | 731.5947 | 14.00 | 0.50 | 2.40E-04 |
| 1.71 | Dichlorodifluoromethane | HMDB0029570 | 118.9282 | 14.02 | 0.50 | 1.90E-07 |
| 1.13 | Ganglioside GM3 (d18:0/26:0) | HMDB0011926 | 1295.9105 | 8.86 | 0.49 | 5.76E-04 |
| 1.26 | 26-Glucosyl-1,3,11,22-tetrahydroxyergosta-5,24-dien-26-oate | HMDB0040405 | 637.3551 | 9.43 | 0.48 | 4.45E-04 |
| 1.37 | PA(O-18:0/21:0) |  | 733.6016 | 14.00 | 0.47 | 1.16E-04 |
| 1.52 | PC(15:0/P-18:1(11Z)) | HMDB0007963 | 730.5806 | 14.00 | 0.47 | 3.84E-05 |
| 1.61 | PS(20:1(11Z)/22:6(4Z,7Z,10Z,13Z,16Z,19Z)) |  | 862.5465 | 10.72 | 0.47 | 5.75E-08 |
| 1.25 | PC(15:0/P-18:0) | HMDB0007962 | 732.5978 | 14.00 | 0.46 | 5.99E-04 |
| 1.12 | CL(i-12:0/18:2(9Z,11Z)/i-12:0/i-12:0) | HMDB0080375 | 1209.8227 | 7.56 | 0.46 | 4.24E-03 |
| 1.29 | TG(16:1(9Z)/16:1(9Z)/20:4(5Z,8Z,11Z,14Z)) | HMDB0005436 | 851.7281 | 0.62 | 0.46 | 7.36E-05 |
| 1.55 | PI(16:0/20:2(11Z,14Z)) | HMDB0009786 | 863.5496 | 10.72 | 0.46 | 1.77E-07 |
| 1.27 | (Z)-1,3-Octadiene | HMDB0061897 | 107.0863 | 8.71 | 0.44 | 4.36E-04 |
| 1.06 | CL(16:0/18:0/20:4(5Z,8Z,11Z,14Z)/20:4(5Z,8Z,11Z,14Z)) | HMDB0056522 | 1476.0269 | 0.62 | 0.44 | 4.26E-03 |
| 1.08 | PC(18:1(9E)/2:0) |  | 562.3515 | 10.19 | 0.44 | 3.33E-03 |
| 2.03 | Traumatic acid | HMDB0000933 | 227.1289 | 6.41 | 0.43 | 7.84E-12 |
| 1.50 | (3b,20R,22R)-3,20,27-Trihydroxy-1-oxowitha-5,24-dienolide 3-glucoside | HMDB0033573 | 633.3273 | 8.17 | 0.42 | 1.65E-05 |
| 1.72 | Ganglioside GA1 (d18:1/24:1(15Z)) | HMDB0004906 | 1335.8804 | 8.16 | 0.42 | 2.57E-07 |
| 1.09 | PC(15:0/20:5(5Z,8Z,11Z,14Z,17Z)) | HMDB0007951 | 764.5213 | 10.21 | 0.42 | 3.20E-03 |
| 1.09 | PE-NMe(15:0/20:4(5Z,8Z,11Z,14Z)) | HMDB0113028 | 738.5079 | 10.21 | 0.42 | 5.07E-03 |
| 1.54 | PI-Cer(t18:0/18:0(2OH)) |  | 842.5774 | 9.82 | 0.41 | 4.22E-04 |
| 1.27 | CDP-DG(16:0/18:0) | HMDB0006969 | 980.5316 | 8.54 | 0.40 | 4.99E-04 |
| 1.66 | PS(DiMe(9,3)/MonoMe(11,3)) | HMDB0061584 | 824.4729 | 10.19 | 0.40 | 1.27E-06 |
| 1.87 | CDP-DG(a-13:0/i-20:0) | HMDB0116076 | 966.5268 | 13.99 | 0.40 | 3.78E-09 |
| 1.57 | 2-Hydroxymyristoylcarnitine | HMDB0013166 | 386.2913 | 9.00 | 0.39 | 4.96E-06 |
| 1.53 | CL(8:0/8:0/12:0/18:2(9Z,11Z)) | HMDB0116916 | 1095.6646 | 8.91 | 0.39 | 1.96E-05 |
| 1.04 | PA(17:1(9Z)/22:2(13Z,16Z)) |  | 739.5111 | 10.21 | 0.38 | 7.93E-03 |
| 1.35 | Donhexocin | HMDB0033087 | 615.4773 | 9.90 | 0.37 | 4.66E-05 |
| 1.02 | CL(a-13:0/i-16:0/18:2(9Z,11Z)/18:2(9Z,11Z))[rac] | HMDB0079068 | 1359.9555 | 9.30 | 0.37 | 6.49E-03 |
| 1.32 | Iocetamic acid |  | 614.8201 | 0.63 | 0.37 | 2.89E-05 |
| 1.14 | Ganglioside GM3 (d18:1/12:0) | HMDB0004842 | 1095.6377 | 8.30 | 0.37 | 6.89E-03 |
| 1.25 | TG(14:0/20:3(5Z,8Z,11Z)/20:5(5Z,8Z,11Z,14Z,17Z)) | HMDB0042447 | 875.7142 | 0.62 | 0.36 | 2.05E-04 |
| 1.33 | Methyl methanethiosulfonate | HMDB0031832 | 126.9875 | 13.98 | 0.36 | 2.22E-04 |
| 1.53 | 1-Octanesulfonic acid |  | 195.1064 | 2.37 | 0.36 | 4.27E-07 |
| 1.53 | PGP(16:1(9Z)/18:1(11Z)) | HMDB0013490 | 825.4763 | 10.19 | 0.36 | 2.24E-05 |
| 1.05 | LacCer(d18:0/20:0) |  | 920.7045 | 0.62 | 0.36 | 1.47E-03 |
| 1.39 | CL(8:0/8:0/14:0/18:2(9Z,11Z)) | HMDB0117061 | 1123.6816 | 8.89 | 0.35 | 7.96E-05 |
| 1.32 | PI(19:0/22:4(7Z,10Z,13Z,16Z)) |  | 927.6124 | 8.90 | 0.34 | 1.42E-03 |
| 1.88 | PIP(16:0/22:5(4Z,7Z,10Z,13Z,16Z)) | HMDB0009937 | 963.5059 | 13.98 | 0.34 | 2.79E-09 |
| 1.25 | PC(24:2(5Z,9Z)/24:2(5Z,9Z))[U] |  | 950.7589 | 0.62 | 0.34 | 9.45E-05 |
| 1.70 | PI(13:0/12:0) |  | 711.4075 | 9.79 | 0.33 | 3.15E-07 |
| 1.77 | Araloside S1 | HMDB0035497 | 1199.6510 | 8.15 | 0.33 | 4.37E-08 |
| 1.24 | PE-NMe(20:2(11Z,14Z)/24:1(15Z)) | HMDB0113365 | 868.6928 | 0.62 | 0.33 | 1.07E-04 |
| 1.91 | PIP(18:2(9Z,12Z)/20:1(11Z)) | HMDB0009989 | 967.5405 | 13.99 | 0.33 | 2.65E-09 |
| 1.88 | Phosphorochloridic acid |  | 114.9339 | 14.02 | 0.32 | 1.33E-09 |
| 1.37 | Cyanoacetic acid |  | 84.0089 | 10.29 | 0.32 | 2.09E-04 |
| 1.63 | GlcCer(d18:1/23:0) |  | 798.6902 | 0.62 | 0.32 | 1.67E-08 |
| 1.36 | Cer(d18:0/14:0) | HMDB0011759 | 512.5108 | 10.02 | 0.32 | 5.08E-05 |
| 1.07 | PA(O-16:0/19:1(9Z)) |  | 675.5320 | 13.99 | 0.31 | 4.02E-03 |
| 1.21 | PE-NMe(13D5/13M5) | HMDB0113797 | 936.6776 | 0.62 | 0.31 | 1.73E-04 |
| 1.37 | PG(P-16:0/20:5(5Z,8Z,11Z,14Z,17Z)) |  | 753.5084 | 7.56 | 0.31 | 3.54E-05 |
| 1.11 | CL(i-13:0/i-22:0/i-12:0/i-16:0) | HMDB0073662 | 1339.9281 | 8.86 | 0.31 | 4.54E-03 |
| 1.39 | Permetin A | HMDB0030527 | 1099.6881 | 9.16 | 0.30 | 8.76E-05 |
| 1.00 | Cytidine 5'-monophosphate-N-acetylneuraminic acid | HMDB0001176 | 615.1564 | 1.04 | 0.30 | 2.62E-03 |
| 1.28 | Malic acid |  | 133.0140 | 0.86 | 0.30 | 1.20E-03 |
| 1.52 | LysoPC(6:0/0:0) | HMDB0029207 | 356.1838 | 6.38 | 0.30 | 5.60E-07 |
| 1.33 | Anhydroamarouciaxanthin B | HMDB0036911 | 577.3694 | 10.23 | 0.29 | 2.55E-04 |
| 1.08 | PE-NMe2(22:2(13Z,16Z)/24:1(15Z)) | HMDB0114494 | 910.7416 | 0.63 | 0.29 | 1.13E-03 |
| 1.06 | CL(i-14:0/i-12:0/i-12:0/i-20:0) | HMDB0075597 | 1269.8737 | 8.64 | 0.28 | 3.00E-03 |
| 1.31 | PS(19:1(9Z)/0:0) |  | 536.2996 | 7.43 | 0.28 | 4.85E-03 |
| 1.89 | Janthitrem F | HMDB0030530 | 644.3579 | 9.25 | 0.27 | 2.91E-09 |
| 1.35 | N-Hydroxy-L-alanine |  | 104.0349 | 0.66 | 0.27 | 1.95E-04 |
| 1.58 | Leukotriene A4 | HMDB0001337 | 317.2123 | 8.70 | 0.27 | 4.78E-06 |
| 1.45 | PS(O-16:0/20:5(5Z,8Z,11Z,14Z,17Z)) |  | 768.5310 | 7.56 | 0.27 | 1.83E-06 |
| 1.16 | PI(17:2(9Z,12Z)/22:6(4Z,7Z,10Z,13Z,16Z,19Z)) |  | 893.5216 | 10.65 | 0.27 | 4.71E-04 |
| 1.37 | LysoPE(0:0/18:3(6Z,9Z,12Z)) | HMDB0011478 | 474.2628 | 7.67 | -0.25 | 1.63E-04 |
| 1.61 | alpha,alpha'-Trehalose 6-palmitate |  | 581.3549 | 8.79 | -0.26 | 2.53E-08 |
| 1.51 | PS(20:2(11Z,14Z)/0:0) |  | 548.2976 | 9.27 | -0.26 | 4.86E-05 |
| 1.35 | PD 145065 |  | 950.5099 | 8.53 | -0.26 | 1.45E-05 |
| 1.08 | PS(14:0/22:4(7Z,10Z,13Z,16Z)) | HMDB0112303 | 782.4980 | 10.20 | -0.26 | 3.42E-03 |
| 1.23 | PGP(22:5(4Z,7Z,10Z,13Z,16Z)/22:5(4Z,7Z,10Z,13Z,16Z)) | HMDB0116493 | 951.5146 | 8.54 | -0.26 | 1.78E-04 |
| 1.55 | Elatoside I | HMDB0031022 | 955.4965 | 14.00 | -0.27 | 8.10E-06 |
| 1.48 | Mucronine D | HMDB0029335 | 662.3924 | 9.04 | -0.27 | 1.34E-06 |
| 1.82 | CL(16:1(9Z)/16:1(9Z)/18:2(9Z,12Z)/22:6(4Z,7Z,10Z,13Z,16Z,19Z)) | HMDB0057515 | 1443.9481 | 8.41 | -0.27 | 2.22E-08 |
| 1.19 | 19,20-DiHDPA | HMDB0010214 | 361.2385 | 8.87 | -0.27 | 1.17E-03 |
| 1.58 | LysoPE(0:0/20:5(5Z,8Z,11Z,14Z,17Z)) | HMDB0011489 | 498.2627 | 7.60 | -0.27 | 4.82E-06 |
| 1.46 | PG(P-20:0/22:6(4Z,7Z,10Z,13Z,16Z,19Z)) |  | 833.5753 | 8.64 | -0.27 | 2.80E-05 |
| 1.13 | Hydroxydestruxin B | HMDB0040135 | 610.3880 | 9.63 | -0.28 | 8.05E-04 |
| 1.37 | PIP(16:0/22:4(10Z,13Z,16Z,19Z)) | HMDB0009922 | 967.5097 | 8.54 | -0.28 | 1.14E-05 |
| 1.63 | Paradisin C | HMDB0040155 | 725.2911 | 5.87 | -0.29 | 1.26E-06 |
| 1.03 | PI(15:0/13:0) |  | 753.4562 | 10.69 | -0.29 | 7.42E-03 |
| 1.35 | Petroselinic acid | HMDB0002080 | 281.2486 | 10.82 | -0.29 | 1.71E-04 |
| 1.45 | Trihexosylceramide (d18:1/9Z-18:1) | HMDB0004878 | 1048.6996 | 8.64 | -0.29 | 3.20E-05 |
| 1.32 | gamma-Crocetin | HMDB0035099 | 355.1916 | 7.73 | -0.29 | 4.57E-04 |
| 1.51 | Gabunamine |  | 691.3907 | 8.88 | -0.30 | 5.17E-07 |
| 1.34 | (3b,6b,8b,12a)-8,12-Epoxy-7(11)-eremophilene-6-angeloyloxy-8,12-dimethoxy-3-ol | HMDB0031964 | 393.2284 | 7.86 | -0.30 | 1.94E-04 |
| 1.74 | Palmitic acid | HMDB0000220 | 255.2329 | 10.69 | -0.30 | 9.53E-08 |
| 1.32 | CDP-DG(16:0/18:1(9Z)) | HMDB0006971 | 980.5549 | 9.20 | -0.31 | 2.69E-05 |
| 1.13 | CL(i-14:0/i-12:0/i-12:0/i-17:0) | HMDB0075592 | 1225.8168 | 8.64 | -0.31 | 2.39E-03 |
| 1.06 | TG(15:0/17:1(9Z)/15:0) |  | 794.7186 | 0.61 | -0.31 | 1.48E-02 |
| 1.42 | N-lactoyl-Methionine | HMDB0062182 | 220.0650 | 3.14 | -0.31 | 1.12E-04 |
| 1.03 | PE(18:0/18:1(9Z))[U] |  | 744.5528 | 10.20 | -0.32 | 1.48E-02 |
| 1.54 | (9S,10S)-10-hydroxy-9-(phosphonooxy)octadecanoate | HMDB0059632 | 395.2203 | 9.93 | -0.32 | 1.09E-05 |
| 1.42 | Ganglioside GM3 (d18:0/24:0) | HMDB0011923 | 1267.8236 | 9.34 | -0.32 | 5.44E-06 |
| 1.42 | Dynorphin A 9-17 | HMDB0012934 | 1182.6111 | 8.26 | -0.32 | 1.12E-04 |
| 1.09 | CL(i-12:0/18:2(9Z,11Z)/a-13:0/18:2(9Z,11Z))[rac] | HMDB0080448 | 1301.8821 | 8.85 | -0.32 | 4.72E-03 |
| 1.36 | Guggulsterone | HMDB0002726 | 341.2123 | 9.05 | -0.33 | 2.08E-04 |
| 1.01 | PC(24:1(15Z)/24:1(15Z)) | HMDB0008816 | 952.7455 | 0.62 | -0.34 | 1.51E-02 |
| 1.74 | Flumethasone Pivalate | HMDB0014801 | 493.2399 | 14.00 | -0.35 | 3.48E-07 |
| 1.40 | Evasterioside E |  | 717.4418 | 9.45 | -0.35 | 7.13E-06 |
| 1.01 | PI(15:0/22:2(13Z,16Z)) |  | 877.5796 | 8.68 | -0.35 | 1.31E-02 |
| 1.38 | Dihydrofukinolide | HMDB0034662 | 391.2130 | 7.72 | -0.36 | 1.52E-04 |
| 1.04 | CL(i-14:0/i-12:0/i-13:0/18:2(9Z,11Z)) | HMDB0075621 | 1251.8753 | 8.88 | -0.36 | 1.74E-03 |
| 1.75 | Moricizine | HMDB0014818 | 426.1490 | 6.19 | -0.36 | 1.54E-07 |
| 1.46 | LysoPE(0:0/20:2(11Z,14Z)) | HMDB0011483 | 504.3087 | 8.86 | -0.36 | 3.94E-05 |
| 1.53 | 6-{4-[3-(3,7-dimethylocta-2,6-dien-1-yl)-5,7-dihydroxy-6-(4-hydroxy-3-methylbut-2-en-1-yl)-4-oxo-3,4-dihydro-2H-1-benzopyran-2-yl]-3-hydroxyphenoxy}-3,4,5-trihydroxyoxane-2-carboxylic acid | HMDB0129788 | 683.2695 | 6.55 | -0.37 | 9.00E-06 |
| 1.65 | CL(8:0/8:0/13:0/18:2(9Z,11Z)) | HMDB0116977 | 1109.6771 | 8.28 | -0.38 | 1.11E-06 |
| 1.04 | CL(i-14:0/i-12:0/i-12:0/i-19:0) | HMDB0075596 | 1253.8456 | 9.38 | -0.38 | 2.55E-02 |
| 1.17 | Torvoside E | HMDB0041530 | 771.4538 | 9.54 | -0.38 | 3.93E-04 |
| 1.26 | PI(O-18:0/20:3(8Z,11Z,14Z)) |  | 873.5793 | 8.69 | -0.38 | 4.72E-04 |
| 1.60 | S-Carboxymethyl-L-cysteine | HMDB0029415 | 178.0179 | 0.88 | -0.39 | 3.23E-06 |
| 1.70 | Naematolone | HMDB0035781 | 305.1396 | 6.59 | -0.39 | 8.36E-07 |
| 1.39 | Erythrityl Tetranitrate | HMDB0015551 | 303.0057 | 0.62 | -0.39 | 3.93E-05 |
| 1.10 | PA(24:0/24:0) | HMDB0115453 | 871.7364 | 0.62 | -0.40 | 3.98E-03 |
| 1.83 | LysoPE(0:0/22:5(4Z,7Z,10Z,13Z,16Z)) | HMDB0011494 | 526.2942 | 8.50 | -0.40 | 9.01E-09 |
| 1.20 | Phosphate | HMDB0001429 | 96.9693 | 0.80 | -0.41 | 2.33E-03 |
| 1.18 | Ganglioside GA1 (d18:1/26:1(17Z)) | HMDB0004907 | 1365.9193 | 8.77 | -0.41 | 2.53E-04 |
| 1.69 | 3',4',5'-Trimethoxycinnamyl alcohol acetate | HMDB0040891 | 265.1083 | 5.64 | -0.42 | 7.87E-07 |
| 1.03 | CL(16:1(9Z)/16:1(9Z)/16:0/16:1(9Z)) | HMDB0110828 | 1345.8936 | 8.43 | -0.42 | 5.65E-03 |
| 1.24 | CL(i-13:0/i-22:0/i-12:0/i-14:0) | HMDB0073656 | 1311.9601 | 9.31 | -0.45 | 3.08E-04 |
| 1.74 | Dihydrovaltrate | HMDB0034492 | 423.2027 | 6.86 | -0.46 | 1.49E-07 |
| 1.76 | Eicosapentaenoic acid | HMDB0001999 | 301.2174 | 9.69 | -0.46 | 5.85E-08 |
| 1.08 | N-palmitoyl taurine |  | 364.2517 | 6.56 | -0.46 | 1.36E-03 |
| 1.12 | TG(20:3(8Z,11Z,14Z)/20:5(5Z,8Z,11Z,14Z,17Z)/20:5(5Z,8Z,11Z,14Z,17Z))[iso3] |  | 947.6952 | 0.62 | -0.47 | 4.08E-03 |
| 1.23 | PC(20:2(11Z,14Z)/24:1(15Z)) | HMDB0008356 | 894.6906 | 0.62 | -0.47 | 1.38E-03 |
| 1.20 | CL(i-14:0/i-12:0/i-12:0/i-14:0) | HMDB0075588 | 1183.8045 | 8.71 | -0.48 | 1.55E-03 |
| 1.85 | PG(18:3(6Z,9Z,12Z)/0:0) |  | 505.2573 | 8.22 | -0.48 | 9.90E-09 |
| 1.73 | PS(12:0/18:3(6Z,9Z,12Z)) |  | 700.4201 | 10.68 | -0.48 | 1.11E-07 |
| 1.61 | PI(O-16:0/18:2(9Z,12Z)) |  | 819.5447 | 11.86 | -0.48 | 1.95E-06 |
| 1.38 | CDP-DG(18:2(9Z,11Z)/a-25:0) | HMDB0116051 | 1104.6787 | 9.47 | -0.49 | 2.87E-05 |
| 1.01 | Carisoprodol | HMDB0014539 | 261.1813 | 4.51 | -0.49 | 2.52E-03 |
| 1.24 | PI(22:0/22:2(13Z,16Z)) |  | 973.6762 | 0.62 | -0.50 | 2.27E-03 |
| 1.18 | TG(17:1(9Z)/20:4(5Z,8Z,11Z,14Z)/20:4(5Z,8Z,11Z,14Z))[iso3] |  | 913.7201 | 0.62 | -0.51 | 3.18E-03 |
| 1.17 | PS(O-20:0/22:6(4Z,7Z,10Z,13Z,16Z,19Z)) |  | 850.5827 | 11.78 | -0.51 | 3.15E-04 |
| 1.66 | Capsicosin | HMDB0031354 | 1241.5950 | 8.32 | -0.52 | 6.57E-07 |
| 1.19 | PG(12:0/20:5(5Z,8Z,11Z,14Z,17Z)) |  | 713.4463 | 10.09 | -0.53 | 3.02E-04 |
| 1.45 | Thymidine | HMDB0000273 | 241.0832 | 1.79 | -0.56 | 4.76E-05 |
| 1.65 | dicyclohexyl phosphonate |  | 246.1392 | 2.36 | -0.57 | 2.81E-07 |
| 2.07 | MIPC(t18:0/24:0(2OH)) |  | 1086.6823 | 8.42 | -0.57 | 4.33E-13 |
| 1.73 | LysoPC(0:0/16:0) | HMDB0240262 | 494.3254 | 9.93 | -0.58 | 1.20E-07 |
| 1.04 | Cobinamide | HMDB0006902 | 990.5031 | 7.88 | -0.59 | 1.69E-03 |
| 1.27 | Trihexosylceramide (d18:1/26:1(17Z)) | HMDB0004884 | 1162.8340 | 9.94 | -0.59 | 4.00E-04 |
| 1.03 | Glycolic acid |  | 75.0086 | 0.74 | -0.60 | 5.89E-03 |
| 1.77 | Gossypol-polyvinylpyrrolidone |  | 630.2690 | 6.04 | -0.63 | 6.43E-11 |
| 1.87 | L-Tryptophan | HMDB0000929 | 205.0975 | 0.73 | -0.63 | 4.17E-10 |
| 1.48 | 11'-Carboxy-gamma-tocotrienol | HMDB0012518 | 399.2541 | 10.07 | -0.64 | 2.11E-05 |
| 1.97 | PG(18:1(9E)/0:0)[U] |  | 509.2887 | 10.82 | -0.66 | 4.43E-11 |
| 1.25 | PC(22:4(7Z,10Z,13Z,16Z)/P-18:0) | HMDB0008653 | 820.6096 | 8.72 | -0.66 | 6.87E-04 |
| 1.80 | Lablaboside C | HMDB0032893 | 1263.5883 | 8.32 | -0.66 | 1.85E-08 |
| 1.34 | Montelukast | HMDB0014614 | 586.2172 | 5.88 | -0.66 | 1.91E-05 |
| 1.16 | CE(MonoMe(13,5)) | HMDB0061674 | 731.6303 | 10.29 | -0.66 | 1.69E-03 |
| 1.48 | PC(22:6(4Z,7Z,10Z,13Z,16Z,19Z)/22:6(4Z,7Z,10Z,13Z,16Z,19Z)) | HMDB0008748 | 878.5561 | 13.26 | -0.66 | 2.78E-06 |
| 1.98 | (S)-a-Amino-2,5-dihydro-5-oxo-4-isoxazolepropanoic acid N2-glucoside | HMDB0029404 | 333.0938 | 1.08 | -0.67 | 1.20E-10 |
| 1.26 | PE-NMe2(22:4(7Z,10Z,13Z,16Z)/24:1(15Z)) | HMDB0114522 | 904.6818 | 0.62 | -0.68 | 1.35E-03 |
| 1.49 | M(IP)2C(d18:0/18:0) |  | 1212.6337 | 8.30 | -0.68 | 1.16E-04 |
| 2.03 | PS(22:4(7Z,10Z,13Z,16Z)/0:0) |  | 572.2998 | 8.70 | -0.68 | 2.07E-12 |
| 2.12 | Astaxanthin | HMDB0002204 | 595.3801 | 9.48 | -0.69 | 1.11E-13 |
| 1.55 | Ganglioside GM2 (d18:0/24:0) | HMDB0011904 | 1467.9439 | 8.76 | -0.71 | 1.48E-07 |
| 1.37 | PD 166285 |  | 584.1094 | 5.75 | -0.72 | 1.03E-05 |
| 1.17 | TG(12:0/12:0/17:1(9Z))[iso3] |  | 705.6155 | 13.88 | -0.72 | 1.30E-03 |
| 1.13 | CL(16:1(9Z)/16:1(9Z)/16:1(9Z)/22:6(4Z,7Z,10Z,13Z,16Z,19Z)) | HMDB0057485 | 1417.8910 | 8.02 | -0.73 | 2.17E-03 |
| 1.38 | GlcCer(d18:1/26:0) |  | 838.6933 | 0.62 | -0.73 | 3.22E-04 |
| 2.10 | 5,8,11-eicosatrienoic acid |  | 305.2482 | 10.40 | -0.74 | 4.22E-14 |
| 1.57 | PS(18:4(6Z,9Z,12Z,15Z)/18:4(6Z,9Z,12Z,15Z)) |  | 776.4463 | 9.03 | -0.75 | 6.39E-07 |
| 1.24 | Dodecachlorocoronene |  | 706.6191 | 13.87 | -0.75 | 5.90E-04 |
| 1.66 | M(IP)2C(t18:0/16:0(2OH)) |  | 1216.5940 | 8.32 | -0.77 | 1.36E-06 |
| 1.18 | TG(14:0/22:5(4Z,7Z,10Z,13Z,16Z)/O-18:0) | HMDB0042750 | 865.7495 | 9.93 | -0.79 | 1.33E-03 |
| 2.02 | PI(20:5(5Z,8Z,11Z,14Z,17Z)/0:0) |  | 617.2744 | 7.75 | -0.79 | 7.10E-12 |
| 1.82 | Assamsaponin E | HMDB0036316 | 1215.5909 | 8.33 | -0.79 | 1.21E-08 |
| 1.11 | Ganglioside GM2 (d18:1/20:0) | HMDB0004940 | 1412.8314 | 8.33 | -0.79 | 2.35E-03 |
| 1.23 | CL(1'-[14:1(9Z)/14:1(9Z)],3'-[14:1(9Z)/15:1(10Z)])[rac] |  | 1245.8218 | 9.36 | -0.79 | 1.20E-04 |
| 1.66 | PS(DiMe(13,5)/MonoMe(13,5)) | HMDB0061577 | 966.6329 | 9.34 | -0.80 | 7.87E-09 |
| 1.88 | Cycleanine |  | 621.2958 | 8.24 | -0.80 | 1.69E-09 |
| 1.20 | TG(20:0/22:5(4Z,7Z,10Z,13Z,16Z)/O-18:0) | HMDB0046074 | 949.8428 | 9.94 | -0.80 | 1.39E-03 |
| 1.15 | Glc-GP(18:0/20:4(5Z,8Z,11Z,14Z)) |  | 885.5483 | 9.34 | -0.80 | 1.74E-03 |
| 1.76 | PI(20:3(8Z,11Z,14Z)/0:0) |  | 621.3050 | 8.77 | -0.81 | 5.78E-08 |
| 1.92 | Ferrioxamine B | HMDB0240270 | 614.2740 | 6.16 | -0.85 | 6.36E-15 |
| 1.06 | CE(DiMe(11,5)) | HMDB0061668 | 717.6151 | 13.88 | -0.86 | 5.17E-03 |
| 1.83 | PC(O-6:0/0:0)[U] |  | 342.2037 | 7.47 | -0.86 | 1.22E-11 |
| 1.19 | SM(d18:0/23:0) | HMDB0012093 | 803.6777 | 8.53 | -0.87 | 1.54E-03 |
| 1.36 | {[6-(5,7-dihydroxy-4-oxo-2-phenyl-3,4-dihydro-2H-1-benzopyran-8-yl)-3,4,5-trihydroxyoxan-2-yl]methoxy}sulfonic acid | HMDB0124786 | 497.0775 | 1.10 | -0.87 | 1.69E-04 |
| 1.11 | ACEXAMIC ACID |  | 172.0971 | 4.63 | -0.88 | 3.10E-03 |
| 1.10 | PI(P-20:0/21:0) |  | 921.6762 | 8.42 | -0.90 | 1.48E-03 |
| 1.34 | Ganglioside GM2 (d18:1/25:0) | HMDB0004946 | 1482.9697 | 9.88 | -0.94 | 1.92E-05 |
| 1.95 | LysoPI(20:4(5Z,8Z,11Z,14Z)/0:0) | HMDB0061690 | 619.2892 | 8.20 | -0.96 | 1.47E-10 |
| 1.23 | CL(16:1(9Z)/16:1(9Z)/16:1(9Z)/20:4(5Z,8Z,11Z,14Z)) | HMDB0057482 | 1395.9644 | 9.64 | -0.96 | 1.49E-04 |
| 2.12 | 9Z-Eicosenoic acid | HMDB0062436 | 309.2799 | 11.97 | -0.98 | 1.56E-14 |
| 1.62 | Ganglioside GM2 (d18:1/26:1(17Z)) | HMDB0004943 | 1492.9561 | 8.39 | -1.05 | 7.42E-06 |
| 1.67 | PE-NMe2(18:3(6Z,9Z,12Z)/20:5(5Z,8Z,11Z,14Z,17Z)) | HMDB0114113 | 786.5090 | 10.82 | -1.08 | 4.61E-07 |
| 1.94 | alpha-Pyrone-4,6-dicarboxylic acid |  | 185.0075 | 1.42 | -1.09 | 1.89E-14 |
| 1.34 | TG(20:5(5Z,8Z,11Z,14Z,17Z)/22:6(4Z,7Z,10Z,13Z,16Z,19Z)/O-18:0) | HMDB0055800 | 937.7705 | 10.06 | -1.13 | 1.70E-04 |
| 1.44 | DG(8:0/18:0/0:0) | HMDB0092933 | 485.4305 | 9.53 | -1.14 | 2.25E-06 |
| 1.92 | N-butyl Oleate | HMDB0062659 | 337.3113 | 13.86 | -1.14 | 3.76E-10 |
| 1.30 | PGP(22:6(4Z,7Z,10Z,13Z,16Z,19Z)/20:3(5Z,8Z,11Z)) | HMDB0116477 | 925.5007 | 14.02 | -1.15 | 7.02E-05 |
| 1.29 | 3-O-Sulfogalactosylceramide (d18:1/16:0) | HMDB0012313 | 780.5402 | 10.79 | -1.16 | 4.62E-05 |
| 1.96 | PI(22:6(4Z,7Z,10Z,13Z,16Z,19Z)/0:0) |  | 643.2896 | 8.22 | -1.16 | 6.25E-11 |
| 1.06 | N-Nitroethylenediamine | HMDB0031226 | 106.0623 | 2.26 | -1.17 | 1.26E-03 |
| 1.98 | PG(20:2(11Z,14Z)/0:0) |  | 535.3042 | 11.86 | -1.20 | 2.82E-11 |
| 1.96 | PG(22:6(4Z,7Z,10Z,13Z,16Z,19Z)/0:0) |  | 555.2735 | 8.80 | -1.22 | 5.55E-11 |
| 2.07 | 2-Methylglutaric acid | HMDB0000422 | 145.0508 | 1.42 | -1.23 | 2.85E-13 |
| 2.12 | 2-Methylbutanoic acid |  | 101.0607 | 1.42 | -1.24 | 2.00E-14 |
| 2.17 | MIPC(d18:0/20:0) |  | 998.6286 | 8.39 | -1.28 | 7.18E-16 |
| 2.08 | 6-hydroxy-2-hexynoic acid |  | 127.0398 | 1.42 | -1.29 | 1.96E-13 |
| 2.14 | PI(18:2(9Z,12Z)/0:0) |  | 595.2896 | 8.15 | -1.36 | 2.74E-15 |
| 1.98 | PG(18:2(9Z,12Z)/0:0) |  | 507.2735 | 8.86 | -1.44 | 4.24E-11 |
| 1.42 | Digitoxose |  | 147.0660 | 1.16 | -1.52 | 5.27E-05 |
| 1.28 | Cer(d18:0/12:0) | HMDB0011758 | 484.4602 | 9.55 | -1.55 | 1.19E-04 |
| 1.92 | 2-[4,6-Bis(2,4-dimethylphenyl)-1,3,5-triazin-2-yl]-5-(octyloxy)phenol | HMDB0037802 | 508.2985 | 8.65 | -1.61 | 3.66E-10 |
| 2.29 | 4-hydroxy-valeric acid |  | 117.0554 | 2.57 | -1.63 | 1.08E-20 |
| 1.69 | SQMG(16:1(9Z)/0:0) |  | 555.2833 | 8.78 | -1.70 | 2.12E-09 |
| 1.63 | Phenylglyoxylyl-CoA |  | 898.1171 | 1.03 | -1.90 | 3.27E-06 |
| 2.00 | 1-Stearoylglycerophosphoglycerol | HMDB0061697 | 511.3043 | 13.97 | -1.98 | 1.29E-11 |
| 1.68 | Kiwiionoside | HMDB0038691 | 407.2276 | 1.02 | -1.99 | 1.72E-06 |
| 1.13 | N-Stearoylsphingosine | HMDB0000829 | 566.5510 | 10.17 | -4.46 | 9.49E-04 |


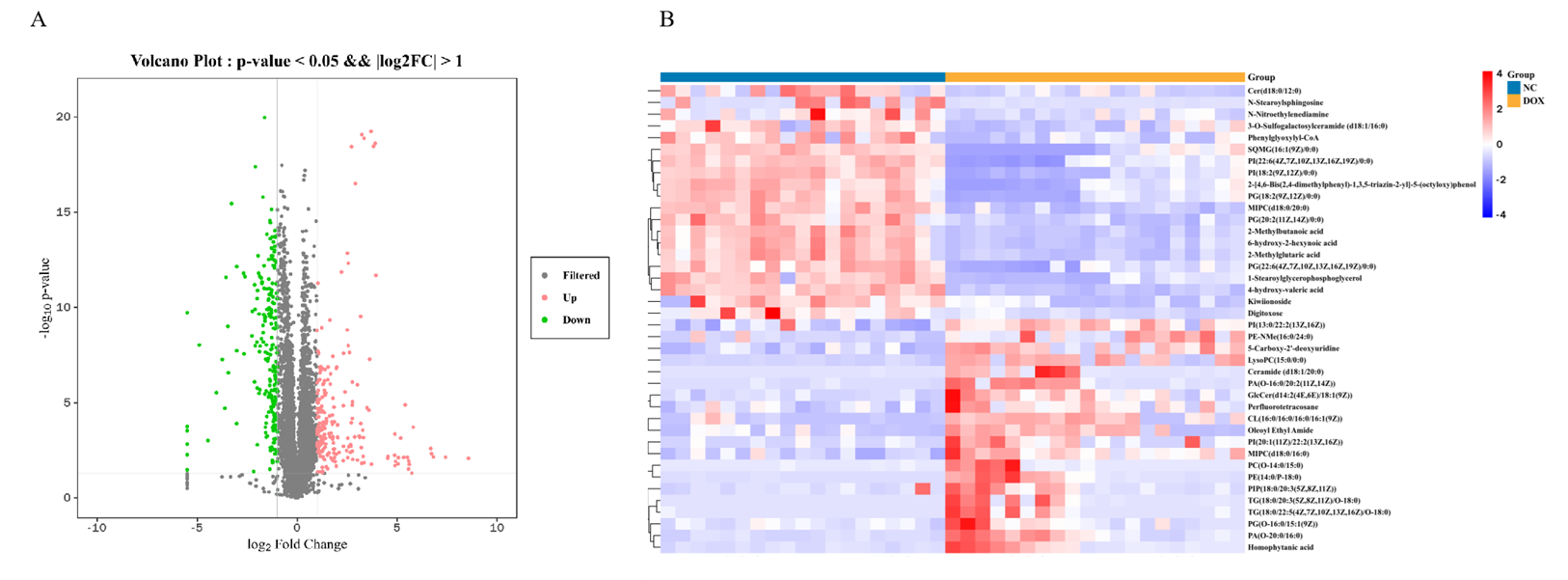


Figure S1 Differential Metabolite Analysis Between DOX-Treated and Control Groups; (A),The volcano plot visualizes the magnitude and statistical significance of metabolite changes between DOX-treated and control groups.X-axis: Log_2_ fold change (DOX/Control), with positive values indicating upregulation and negative values indicating downregulation in DOX-treated samples.Y-axis: -log₁₀(p-value) derived from a two-tailed Student’s t-test.Significance thresholds: Metabolites with |log2fold change| >1 and *p* < 0.05 (adjusted) are highlighted in red (upregulated) and green (downregulated).Gray dots: Non-significant metabolites. (B), The heatmap displays the relative abundance patterns of TOP 40 significantly altered metabolites (|fold change| >1, p < 0.05) across DOX-treated and control samples.
